# Incidence and risk factors of hospitalisations for respiratory syncytial virus among children aged less than 2 years

**DOI:** 10.1017/S0950268822000152

**Published:** 2022-02-02

**Authors:** Wasef Na'amnih, Eias Kassem, Shebly Tannous, Viktoria Kagan, Athar Jbali, Elizabeth Hanukayev, Sarit Freimann, Uri Obolski, Khitam Muhsen

**Affiliations:** 1 Department of Epidemiology and Preventive Medicine, School of Public Health, Sackler Faculty of Medicine, Tel Aviv University, Tel Aviv 6997801, Israel; 2 Department of Paediatrics, Hillel Yaffe Medical Center, Hadera 38100, Israel; 3 Porter School of Environmental and Earth Sciences, Raymond and Beverly Sackler Faculty of Exact Sciences, Tel Aviv University, Tel Aviv, Israel

**Keywords:** Hospitalisation, incidence, respiratory syncytial virus, risk factors, seasonality

## Abstract

The study aim was to examine the incidence and risk factors of respiratory syncytial virus (RSV) bronchiolitis hospitalisations and disease severity among infants. We compared demographic and health characteristics of children aged 0–23 hospitalised for RSV bronchiolitis (cases, *n* = 1227) during 2008–2018 and control children (*n* = 554) of the same age admitted for non-respiratory disease. RSV antigen was detected in nasal swabs by immunochromatography. Multiple logistic regression models were applied. The average annual incidence of hospitalisation for RSV bronchiolitis was 12.6 per 1000 and 1.7 per 1000 (*P* < 0.001) among infants and toddlers, respectively, with winter seasonality (November–March). The risk of hospitalisation for RSV bronchiolitis increased among children aged 0–5 months (OR 7.66; 95% CI 5.61–10.45) and 6–11 months (OR 12.88, 95% CI 8.48–19.55), compared to those aged 12–23 months. Additional risk factors were living in low *vs.* higher socio-economic status towns (OR 1.49; 95% CI 1.14–1.95), having chronic medical conditions (OR 2.75; 95% CI 1.61–4.70), birth month (October–January *vs.* June–September) (OR 2.19; 95% CI 1.60–2.99) and history of stay in neonatal intensive care unit at birth (OR 2.37; 95% CI 1.27–4.41). Male children and those who had pneumonia were more likely to have severe RSV bronchiolitis. In conclusion, the burden of hospitalisations for RSV bronchiolitis is high, especially in young infants. Effective preventive measures such as RSV active vaccines can reduce the risk of hospitalisations for RSV bronchiolitis among these vulnerable groups.

## Introduction

Respiratory syncytial virus (RSV) is a main cause of acute bronchiolitis [1], and pneumonia in infants and young children [2, 3]. Usually the incidence of RSV bronchiolitis displays a seasonal pattern, during the cold (winter) months in regions with temperate climates [4]. Globally, the highest incidence of severe RSV lower respiratory tract infection was reported among children aged less than 6 months [2, 5, 6]. The youngest age groups at the beginning of RSV season have increased risk for RSV hospitalisation [7, 8], and the severity of RSV disease decreases with age [9]. The risk for RSV hospitalisation was shown to be increased among premature born children, those having a chronic lung disease, congenital heart disease (CHD), cerebral palsy, Down syndrome or immunosuppression conditions [10–14], although it was shown that the majority of RSV-related hospitalisation occur among children lacking these risk factors [10]. Therefore, the identification of potential risk factors for RSV bronchiolitis should be perused. Indeed, a systematic review has highlighted the need for further studies to better identify, update and estimate risk factors associated with hospitalisation for RSV bronchiolitis [15].

Palivizumab is a monoclonal antibody that provides passive immunisation against RSV in high-risk infants less than 2 years of age, which should be given at the start of the RSV season [16]. The 2014 Guidance of the American Academy of Paediatrics for RSV Prophylaxis recommends the administration of a maximum 5 monthly doses (15 mg/kg/dose) of palivizumab during RSV season, during the first year of life to infants born at a gestational age of <29 weeks 0 days' gestation, infants with chronic lung disease of prematurity (defined as preterm infants <32 weeks 0 days' gestation who require >21% oxygen for at least the first 28 days of life), or infants with hemodynamically significant CHD, all should be less than 12 months of age at the start of the RSV season. In the second year of life, palivizumab prophylaxis is recommended only for children who satisfy the definition of chronic lung disease of infancy and continue to require supplemental oxygen, chronic corticosteroid therapy or diuretic therapy within 6 months of the onset of the second RSV season [17, 18].

A recent census of expert group from Israel, Europe and Canada recommended palivizumab for the following groups: preterm infants (<29-week gestational age and ≤9 months at the start of the RSV season; 29–31-week gestational age and ≤6 months at the start of the RSV season and 32–35-week gestational age and high-risk), preterm born children aged ≤24 months with chronic lung disease/bronchopulmonary dysplasia, children aged ≤24 months with significant CHD; and other high-risk populations, such as Down Syndrome, pulmonary/neuromuscular disorders, immunocompromised and cystic fibrosis [19].

Multiple RSV vaccine candidates are currently in development, and some vaccines for infant use are in clinical trials [20, 21], but so far there is no licenced vaccine against RSV.

Previous studies from Israel on RSV bronchiolitis among children [22–30] were mostly descriptive studies of limited duration and did not include a comparison group of children without bronchiolitis, thus evidence on the risk factors remains elusive. Moreover, information on the population size (denominators) was lacking in most studies, therefore most of these studies lacked estimates of incidence rates of RSV bronchiolitis; thus evidence on the disease burden is limited. Some of these studies were conducted in tertiary hospitals or focused on severe RSV bronchiolitis, thus might not represent well the full spectrum of patients. Accordingly, better understanding of the incidence and risk factors of RSV bronchiolitis is needed. To address these gaps, the aim of the current study was to determine the incidence rates and demographic and clinical risk factors of RSV bronchiolitis-associated hospitalisations among children aged 0–23 months, in northern Israel during 2008–2018. Additionally, we examined the correlates of severe RSV disease.

## Methods

### Study design and population

A retrospective cohort study was conducted at Hillel Yaffe Medical Center in Hadera in Israel, using hospitalisation records of children aged 0–23 months, during the period 1 January 2008 to 30 June 2018. Hillel Yaffe Medical Centre is a 515-bed hospital located at Hadera sub-district. Based on 2018 estimates, about 450 000 people live in this region, 56.1% Jews and 43.9% Arabs [31]. The respective numbers of live births in 2018 were 4226 and 3955 [31]. We assessed the risk factors of RSV bronchiolitis, in a case-control study in which we reviewed medical records of cases aged 0–23 months hospitalised for bronchiolitis with laboratory verification of RSV and a control group of children from the same age group hospitalised due to trauma (fractures and falls) during the same period. The independent variables included socio-demographics: the child's age (in months) on hospitalisation; sex (male/female); and population group (Arabs or Jews). We also assessed calendar month of birth as an independent variable, since it was shown to be associated with RSV risk [32]. Socio-economic status (SES) was determined based on SES rank of the town of residence that is defined by the Israel Central Bureau of Statistics [33]. Towns with SES ranks of 1–4 and 5–10 were classified as low and high SES communities, respectively. Additional independent variables included the following medical conditions: emergency room visits in the month prior to the current hospitalisation (yes *vs.* no); hospitalisation in the month before the current hospitalisation (yes *vs.* no); past hospitalisations (yes *vs.* no); birth weight (in grams); gestational age at birth (in weeks) and stay in neonatal intensive care after birth (yes *vs.* no). We also assessed the history of congenital malformations (yes *vs.* no) and having chronic medical conditions (yes *vs.* no), including wheezing/asthma, gastro-oesophageal reflux disease, Down syndrome and anaemia. We also collected information on the therapies that the child received for the treatment of the current episode of RSV bronchiolitis, including inhalations, steroids, oxygen, etc.

The severity of RSV bronchiolitis was assessed using a summative disease severity score that was constructed based on having the following factors: dyspnoea, tachypnoea, hypoxia (oxygen saturation in room air<92%), cough, fever and length of hospitalisation greater than median (3 days). For each factor, the child was ‘accredited’ one point. Children with the median score or above were classified as having a severe illness, otherwise they were classified as having less severe disease [30].

### Laboratory methods

Nasal swabs were taken from children hospitalised due to a clinical diagnosis of bronchiolitis and tested for RSV antigen utilizing an immunochromatographic assay; BinaxNOW (Alere, Maine, USA). Sensitivity and specificity of this kit were estimated at 90–94% and 100%, respectively, compared to viral culture and/or reverse transcription polymerase chain reaction assay [34, 35]. RSV testing practices were similar during the study period.

### Statistical analysis

#### Description of the study sample

Categorical variables were described using frequencies and percentages and continuous variables were described using medians, means and standard deviation (SD).

#### Calculation of incidence rates

We obtained information on the population size by year and age group of Hadera sub-district from the Central Bureau of Statistics [31]. We estimated that 80–90% of the population in Hadera sub-district receives hospitalisation services from Hillel Yaffe Medical Centre. The size of the population aged *<*24 months was estimated as 40% of the population aged 0–4 years living in Hadera sub-district. The average population aged <24 months was 15 674 between 2008 and 2018 [31] (Table S1). The annual incidence rate of laboratory-confirmed cases of RSV bronchiolitis was calculated using the annual number of RSV laboratory-confirmed cases in the numerator and population size of children aged 0–23 months served by Hillel Yaffe Medical Centre in the denominator each year. We also calculated the average incidence rate during the study period. The incidence of hospitalisation for RSV bronchiolitis (per 1000 children) and exact 95% confidence intervals (CIs), based on a Poisson distribution for the incidence rates, were calculated. A similar approach was followed for the calculation of age-specific and population group-specific incidence rates.

We examined trends in the overall incidence rate using Cochrane-Armitage test for linear trend, and calculated the annual percent change from regression equation using the slope coefficient for the log-transformed annual data. Serial correlation of residuals across annual incidence rates was assessed using Run test, which was not significant (two-tailed *P* > 0.2).

RSV seasonality was described on an annual basis using a calendar year and month of hospitalisation starting on July and ending on June of the following year (Table S1).

#### Risk factors

Differences between cases and controls in potential risk factors were examined using the *χ*^2^ test or Fisher exact test for categorical variables and Student's *t* test for continuous variables and Mann–Whitney *U* test for variables with skewed distribution. Multivariable analysis was performed using logistic regression models. Factors that were associated with RSV bronchiolitis with *P* *<* 0.05 in bivariate analysis were included in the multivariable analysis in a stepwise manner. Since the variables population group and residential SES were highly correlated (Phi coefficient 0.82, *P* *<* 0.001), they were analysed in separate models, we present both models with parameters of model fit such as Nagelkerke *R*^2^ and Akaike's information criterion (AIC). We examined differences in disease severity according to demographic and clinical factors using the *χ*^2^ test and multivariable logistic regression models. Odds ratios (ORs) and 95% CIs for each variable were obtained from logistic regression models. Statistical significance was set at *P* *<* 0.05. Data were analysed using SPSS version 27 (IBM, Armonk, New York, USA) and Winpepi software.

### Ethical approval

The study protocol was approved by the Helsinki committee of Hillel Yaffe Medical Centre. An exemption of signing an informed consent was given by Helsinki committee given the retrospective study design.

## Results

Overall, 1227 children aged 0–23 months (57.4% males) were hospitalised for RSV bronchiolitis at Hillel Medical Centre during the study period. The male *vs.* female ratio was 1.35. The median age of children hospitalised for RSV hospitalisations was 3 months. Among the cases, 64.1% aged 0–5 months, 24.2% aged 6–11 months and 11.7% aged 12–23 months. None of the patients with RSV bronchiolitis died during hospitalisation, one patient needed mechanical ventilation and was transferred to the intensive care unit, and the rest were discharged in good health. The median length of hospital stay was 3 days (range 1–34). Overall 97 patients (7.9%) were eligible for palivizumab immunoprophylaxis according to the Israeli Ministry of Health, of these 11 (11.3%) had documentation for the recipient of palivizumab.

### Incidence rates

**Fig. 1.**
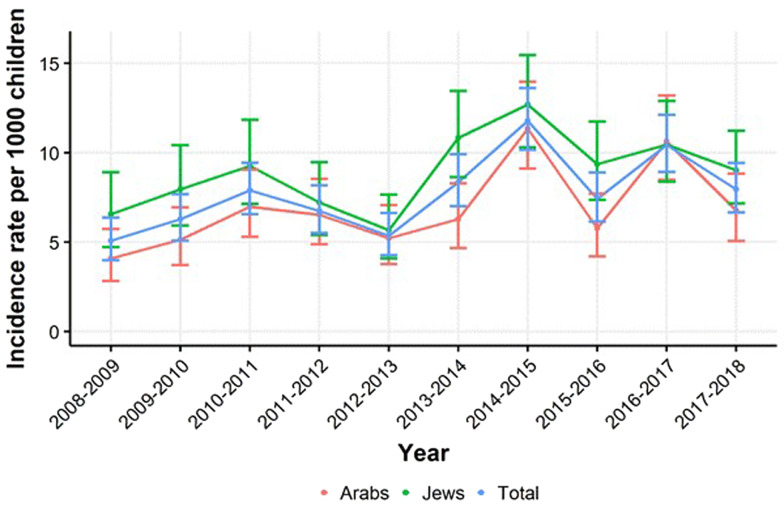
Incidence rates and 95% confidence intervals of respiratory syncytial virus hospitalisations in children aged 0–23 months by population group, Hadera sub-district, Israel, 2008–2018.

The annual incidence rates of hospitalisation for RSV bronchiolitis during 2008–2018 are presented in Figure 1. The incidence rates of hospitalisations for RSV bronchiolitis increased from 5.1 per 1000 children in 2008 to 8.0 per 1000 children in 2018. The Cochrane-Armitage test for linear trend was significant (*P* < 0.001). The annual percent increase was 5.87% (95% CI 1.30–10.66%). The average incidence hospitalisation for RSV bronchiolitis between 2008 and 2018 was 7.8 per 1000 children aged 0–23 months, and it was higher among infants aged 0–11 months (12.6 per 1000) than toddlers aged 12–23 months (1.7 per 1000) (*P* < 0.001). The average incidence rate was 1.3-fold higher among Jewish children (9.1 per 1000) compared to Arab children (6.9 per 1000) (*P* < 0.001).

### Seasonality

**Fig. 2.**
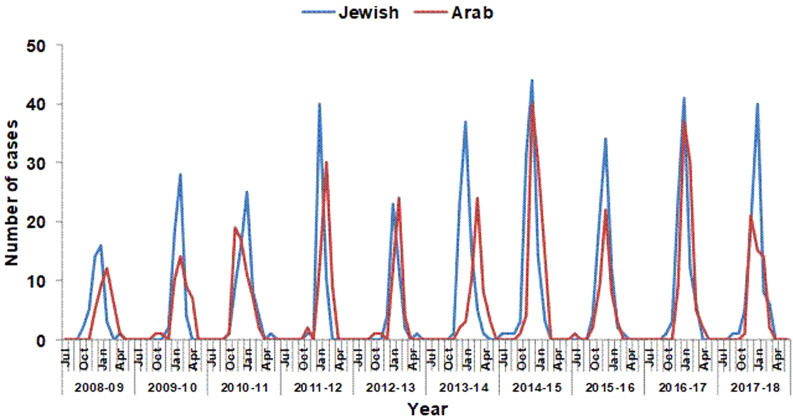
Number of monthly hospitalisations of respiratory syncytial virus bronchiolitis among children aged 0–23 months by population group, Hadera sub-district, Israel 2008–2018.

Typical seasonality was observed in hospitalisations due to RSV bronchiolitis, with an increase that began in October, peaked in January and decreased thereafter (Fig. 2). Considering all years, 96% (1171/1219) of the hospitalisations for RSV bronchiolitis occurred between November and March.

### Risk factors

The percentage of children aged 0–5 months was higher among children with RSV (64.1%) than control children (35.4%) (*P* < 0.001). In addition, 50.2% of children with RSV lived in low SES towns compared to 40.3% among the controls (*P* *<* 0.001). The proportions of Arab children and males were significantly higher among children with RSV bronchiolitis than the control group (*P* < 0.001; *P* = 0.01, respectively). Fifty-three percent of the children with RSV were born in October–January *vs.* 32.5% of those in the control group (*P* < 0.001). The proportions of children with congenital malformations, chronic medical conditions, hospitalisation during the month before current hospitalisation and history of stay in neonatal intensive care unit at birth were significantly higher among children with RSV bronchiolitis than the control group (Table 1). No significant differences between the groups were found in birth weight, gestational age at birth, past hospitalisations and emergency room visit in the last month (Table 1).

**Table 1.** Characteristics of cases hospitalised for RSV-bronchiolitis and controls^a^

Variable	Cases (*N* = 1227)	Controls (*N* = 554)	*P* value^b^
	Number (%)	Number (%)	
Sex			0.01
Male	705 (57.5)	282 (50.9)	
Female	522 (42.5)	272 (49.1)	
Population group			<0.001
Arabs	552 (45.0)	196 (35.4)	
Jews	675 (55.0)	358 (64.6)	
Age, months			
0–5	786 (64.1)	195 (35.4)	<0.001
6–11	297 (24.2)	94 (17.0)	
12–23	144 (11.7)	263 (47.6)	
Socio-economic status of place of residence			<0.001
Low (1–4)	614 (50.2)	220 (40.3)	
High (5–10)	609 (49.8)	326 (59.7)	
Calendar month of birth			<0.001
October–January	648 (52.8)	180 (32.5)	
February–May	226 (18.4)	196 (35.4)	
June–September	353 (28.8)	178 (32.1)	
Median weight at birth (grams) (min-max)	3145 (700–5505)	3175 (660–4800)	0.2
Missing	267	245	
Gestational age at birth (weeks)			0.6
≤36	128 (13.9)	20 (15.6)	
>37	793 (86.1)	108 (84.4)	
Congenital malformations			<0.001
No	1188 (96.8)	272 (100.0)	
Yes	39 (3.2)	0 (0.0)	
Chronic medical conditions			0.01
No	1109 (90.4)	521 (94.0)	
Yes	118 (9.6)	33 (6.0)	
Hospitalisation in the last month			0.005
No	1155 (94.4)	480 (97.6)	
Yes	69 (5.6)	12 (2.4)	
Past hospitalisations			0.07
No	1050 (85.8)	413 (82.3)	
Yes	174 (14.2)	89 (17.7)	
Emergency room visit in the last month			0.3
No	1154 (94.2)	473 (95.6)	
Yes	71 (5.8)	22 (4.4)	
History of stay in neonatal intensive care			0.006
No	881 (92.2)	455 (96.0)	
Yes	75 (7.8)	19 (4.0)	

^a^RSV, respiratory syncytial virus.

^b^*P* value obtained by the *χ*^2^ test or Fisher exact test where appropriate.

In a multivariable analysis (Model 1, Table 2), children aged 0–5 months (OR 7.66, 95% CI 5.61–10.45) and 6–11 months (OR 12.88, 95% CI 8.48–19.55) were at increased risk for RSV bronchiolitis than toddlers aged 12–23 months. Living in low SES towns *vs.* high SES towns was associated with increased risk for RSV bronchiolitis (OR 1.49, 95% CI 1.14–1.95), as well as having a chronic medical conditions (OR 2.75, 95% CI 1.61–4.70), and stay in neonatal intensive care unit at birth (OR 2.37, 95% CI 1.27–4.41). Compared to children who were born during June–September, those who were born during October–January had increased risk for RSV bronchiolitis (OR 2.19, 95% CI 1.60–2.99) and those who were born during February–May had decreased risk (OR 0.48, 95% CI 0.33–0.68). An additional multivariate model (Model 2) included the same variables, but the variable ‘population group’ replaced the variable ‘residential SES’ and showed similar results, and significant association between population group and RSV bronchiolitis but had slightly preferable (AIC = 122.8 and 118.3 for Models 1 and 2, respectively) (Table 2).

**Table 2.** Multiple logistic regression models for factors associated with RSV among hospitalisations

			Model 1		Model 2	
Variable	Unadjusted OR (95% CI)	*P* value	Adjusted OR (95% CI)	*P* value	Adjusted OR (95% CI)	*P* value
Sex						
Male	1.30 (1.06–1.59)	0.01	1.20 (0.92–1.56)	0.2	1.21 (0.93–1.57)	0.2
Female	Reference		Reference		Reference	
Residential SES						
Low SES (1–4)	1.49 (1.22–1.83)	<0.001	1.49 (1.14–1.95)	0.003		
High SES (5–10)	Reference		Reference		Not included	
Population group						
Arabs	1.49 (1.21–1.84)	<0.001	Not included		1.49 (1.14–1.96)	0.004
Jews	Reference				Reference	
Age, months	df = 2		df = 2	<0.001	df = 2	<0.001
0–5	7.36 (5.69–9.52)	<0.001	7.66 (5.61–10.45)	<0.001	7.67 (5.63–10.46)	<0.001
6–11	5.77 (4.24–7.86)	<0.001	12.88 (8.48–19.55)	<0.001	13.00 (8.56–19.73)	<0.001
12–23	Reference		Reference		Reference	
Birth month	df = 2	<0.001	df = 2	<0.001	df = 2	<0.001
October–January	1.82 (1.42–2.32)	<0.001	2.19 (1.60–2.99)	<0.001	2.18 (1.60–2.96)	<0.001
February–May	0.58 (0.45–0.76)	<0.001	0.48 (0.33–0.68)	<0.001	0.46 (0.32–0.67)	<0.001
June–September	Reference		Reference		Reference	
Chronic medical conditions						
No	Reference		Reference		Reference	
Yes	1.68 (1.13–2.51)	0.01	2.75 (1.61–4.70)	<0.001	2.83 (1.66–4.81)	<0.001
Hospitalisation in the last month						
No	Reference		Reference		Reference	
Yes	2.39 (1.28–4.45)	0.006	1.20 (0.56–2.56)	0.6	1.18 (0.55–2.53)	0.7
Stay in neonatal intensive care at birth						
No	Reference		Reference		Reference	
Yes	2.04 (1.22–3.42)	0.007	2.37 (1.27–4.41)	0.006	2.39 (1.28–4.45)	0.006

AIC, Akaike's information criterion; CI, confidence interval; OR, odds ratio; RSV, respiratory syncytial virus; SES, socio-economic status.

Nagelkerke *R*^2^ model 1 = 0.315, AIC = 122.8; Nagelkerke *R*^2^ model 2 = 0.317, AIC = 118.3.

Model 1 included the following independent variables: sex, residential SES, age, birth month, chronic medical conditions, hospitalisation in the last month and history of stay in neonatal intensive care at birth. Model 2 included the same variables as in model 1, but the variable ‘population group’ replaced the variable ‘residential SES’.

### Factors associated with the severity of RSV bronchiolitis

A positive association was found between child's age and the severity of RSV bronchiolitis (*P* for trend <0.001). The proportion of males was higher among children with severe disease *vs.* those with less severe disease (59.4% *vs.* 53.3%; *P* = 0.04), as well as the proportion of children chronic medical conditions (10.8% *vs.* 7.1%; *P* = 0.04), history of stay in neonatal intensive care unit at birth (9.2% *vs.* 5.5%; *P* = 0.04) and having a diagnosis of pneumonia. Treatment with inhalations, steroids, oxygen and vapotherm was more common among children with severe disease than those with less severe disease (Table 3). In a multivariable logistic regression model, being a male and having pneumonia (confirmed by chest X-ray) remained significantly associated with an increased risk for severe RSV bronchiolitis, while children aged 0–5 months had lower risk for severe disease than toddlers aged 12–23 months. Treatment of the current episode with inhalation before hospitalisation, and treatment with oxygen and steroids during hospitalisation were significantly positively associated with disease severity (Table 4).

**Table 3.** Factors associated with RSV bronchiolitis severity

Variable	Severe disease (*N* = 831)	Less severe disease (*N* = 396)		
	*N* (%)	*N* (%)	OR (95% CI)	*P* value
Residential SES				
Low SES (1–4)	406 (48.9)	208 (52.9)	0.85 (0.67–1.08)	0.2
High SES (5–10)	424 (51.1)	185 (47.1)	Reference	
Sex				
Male	494 (59.4)	211 (53.3)	1.28 (1.01–1.64)	0.04
Female	337 (40.6)	185 (46.7)	Reference	
Age, months			df = 2	<0.001
0–5	501 (60.3)	285 (72.0)	0.46 (0.30–0.71)	<0.001
6–11	216 (26.0)	81 (20.5)	0.70 (0.44–1.13)	0.1
12–23	114 (13.7)	30 (7.5)	Reference	
Gestational age at birth (weeks)				
≤36	91 (14.4)	37 (12.9)	0.88 (0.59–1.33)	0.5
>36	543 (85.6)	250 (87.1)	Reference	
Chronic medical conditions^a^	90 (10.8)	28 (7.1)	1.60 (1.03–2.48)	0.04
Chest X-ray				
Pneumonia	527 (63.4)	162 (41.1)	2.48 (1.94–3.17)	<0.001
No pneumonia^b^	304 (36.6)	232 (58.9)	Reference	
History of stay in neonatal intensive care at birth^a^	56 (9.2)	19 (5.5)	1.73 (1.01–2.97)	0.04
History of respiratory disorders^a^	25 (10.2)	2 (3.6)	3.01 (0.69–13.11)	0.1
Congenital malformations^a^	28 (3.4)	11 (2.8)	1.22 (0.60–2.48)	0.6
Treatment with inhalations during hospitalisation^a^	714 (86.1)	320 (81.2)	1.44 (1.04–1.98)	0.03
Treatment with inhalations before hospitalisation^a^	302 (37.8)	114 (29.2)	1.47 (1.14–1.91)	0.003
Oxygen therapy^a^	382 (47.7)	74 (19.1)	3.86 (2.89–5.15)	<0.001
Steroid therapy during hospitalisation^a^	222 (27.4)	35 (9.0)	3.80 (2.60–5.56)	<0.001
Steroid therapy before hospitalisation^a^	156 (19.5)	65 (16.5)	1.23 (0.89–1.69)	0.2
Vapotherm therapy^a^	38 (6.5)	7 (2.1)	3.30 (1.46–7.47)	0.003

CI, confidence interval; OR, odds ratio; RSV, respiratory syncytial virus; SES, socio-economic status.

a Reference category = not having the condition.

b No pneumonia by chest X-ray or by clinical judgment (i.e. chest X-ray was not ordered).

**Table 4.** Multiple logistic regression model for factors associated with RSV severity

Variable	Adjusted OR (95% CI)	*P* value
Age, months	df = 2	0.1
0–5	0.56 (0.33–0.96)	0.04
6–11	0.68 (0.38–1.23)	0.2
12–23	Reference	
Sex		
Male	1.43 (1.07–1.91)	0.02
Female	Reference	
History of stay in neonatal intensive care at birth		
Yes	1.23 (0.60–2.53)	0.5
No	Reference	
Chest X-ray-pneumonia (yes *vs.* no)^a^		
Yes	1.89 (1.41–2.55)	<0.001
No	Reference	
Chronic medical conditions		
Yes	1.12 (0.67–1.86)	0.7
No	Reference	
Treatment with inhalations during hospitalisation		
Yes	1.06 (0.67–1.68)	0.8
No	Reference	
Treatment with inhalations before hospitalisation		
Yes	1.49 (1.09–2.03)	0.01
No	Reference	
Oxygen therapy during hospitalisation		
Yes	2.78 (1.94–3.97)	<0.001
No	Reference	
Steroid therapy during hospitalisation		
Yes	2.46 (1.51–3.99)	<0.001
No	Reference	
Vapotherm therapy during hospitalisation		
Yes	1.65 (0.68–3.98)	0.3
No	Reference	

CI, confidence interval; df, degrees of freedom; OR, odds ratio; RSV, respiratory syncytial virus; SES, socio-economic status.

Nagelkerke *R*^2^ = 0.179.

a No pneumonia by chest X-ray or by clinical judgment (i.e. chest X-ray was not ordered).

## Discussion

Herein we described the incidence rates and risk factors of RSV bronchiolitis-associated hospitalisations in children aged 0–23 months. The average incidence rate of hospitalisations for RSV bronchiolitis was 7.8 per 1000 children per year. In a previous study [30] in the same population, we reported an average incidence for RSV hospitalisations of 5.7 per 1000 children during 2008–2011.

The incidence rate of RSV bronchiolitis was higher among infants than toddlers, an average annual rate of 12.6 per 1000 *vs.* 1.7 per 1000. This finding is comparable to the incidence rates reported in a nationwide study based on diagnoses codes, which showed average rates of 12.2 per 1000 and 1.2 per 1000 in the respective age groups during 2000–2017 [26]. Other international studies [36–40] reported similarly higher incidence of RSV bronchiolitis among infants than toddlers, although the rates were higher in Denmark and the United States than our estimate. For example, a study from Denmark reported high incidence rates of 29.4, among children 0–11 months compared to 6.3 per 1000 among children aged 12–23 months [36]. In the United States, the corresponding rates were 13.4 and 5.0 per 1000 children [39]. We found that the highest risk for RSV bronchiolitis was among children aged less than 6 months, which is in agreement with the nationwide analysis from Israel [25] and other reports showing that 49–70% of cases fall in this age category [37, 41]. Moreover, as we and others have previously shown [42–44], an additional adverse outcome of RSV infections is a substantial risk for unnecessary antibiotic treatment, administered to about 25–50% of RSV patients.

Collectively these findings highlight the need to develop an effective vaccine that provides durable protection against RSV infection at least during the first few months of life. Such vaccine candidate might be an active vaccine given shortly after birth or a maternal vaccine given during late pregnancy anticipating placental transfer of anti-RSV maternal IgG antibodies to the foetus [45–47]. A recent study showed no protective effect of increased level of cord-blood RSV-neutralizing antibodies against acute RSV infection during the first 6 months of life, although protection was evident at age 12–24 months [48]. A randomised controlled phase III clinical trial with RSV fusion protein nanoparticle vaccine, which included 4579 pregnant women, showed no significant protection against the prespecified endpoint for the efficacy of RSV-associated, medically significant lower respiratory tract infection up to age 90 days after vaccination (vaccine efficacy was 39.4%) [20]. It was postulated that the lack of statistical power might explain the findings of this trial [47]. However, there was an indication of potential protection conferred by maternal immunisation with respect to other end-point events related to RSV respiratory disease in infants [20]. Additional clinical trials with other vaccines and larger sample size with maternal RSV vaccines will likely provide answers regarding the relevance of maternal RSV immunisation for the prevention of RSV disease during the first few months of life (reviewed by Gunatilaka and Giles) [47]. Possibly, the combination of maternal vaccination and child vaccination with an active RSV vaccine will be needed, to provide both early and durable protection against RSV disease during infancy.

Some studies conducted in the United States showed that children from minority groups and those in low SES had higher rates of bronchiolitis from RSV compared to the general population [49, 50]. However, we found that the incidence rate was 1.3-fold higher among Jewish children compared to Arab children. The Arab population in Israel is the main ethnic minority. Interestingly, another study conducted in the southern Israel showed a higher incidence rate of RSV bronchiolitis among Bedouin children than Jewish children [26], which might be explained by the lower SES of the Bedouin population in this region compared to the general Arab population.

RSV seasonality in our study was consistently observed during the cold fall-winter months of November–March, in agreement with prior reports [4, 32, 36, 41, 51–54]. Multiple meteorological factors were shown to be associated with RSV incidence rates and activity [53–55]. Temperature was inversely related to RSV incidence, while relative humidity, air pressure and cloud cover were shown to positively correlate with RSV incidence [54]. RSV is transmitted via close contact and respiratory droplets; therefore, these meteorological conditions might contribute to the virus stability and survival in the environment and enhance the virus transmission during the cold months. Behavioural factors such as social mixing are also expected to be necessary for RSV transmission. This assumption was recently supported by changes in RSV seasonal circulation in Israel during COVID-19 pandemic [56]. During the winter of 2020–2021, Israel was under a lockdown due to surge in COVID-19 [57], a period that was characterised by low level of RSV disease incidence. Around March 2021 after lifting of the lockdown, an increase in RSV activity was observed [56], thus highlighting the importance of behavioural factors in the transmission of RSV.

An interesting finding of our study is the significant association between the calendar month of birth and RSV bronchiolitis. Children who were born on October, November, December and January were at a higher risk for RSV bronchiolitis compared to those who were born on June to September. A study conducted in northern Spain [32] also showed that being born on October to December was associated with increased risk of hospitalisation for RSV bronchiolitis. A possible explanation of the positive association between birth month during October–January and the risk of RSV bronchiolitis is that children born in these months actually spend their few first months of life during the season of RSV activity, thus having more chances to encounter the virus.

A history of stay in a neonatal intensive care unit after birth was associated with an increased risk for RSV bronchiolitis. Stay in a neonatal intensive care unit is determined by medical conditions of the newborn, including gestational age at birth. Interestingly in our study, there was no significant association between gestational age at birth or birth weight with the risk of RSV bronchiolitis. This implies that medical conditions that require treatment and observation in intensive care unit at birth are more influential factors of RSV risk than birth weight/gestational age *per se*. Other studies, however, have shown increased risk for hospitalisation due to RSV bronchiolitis in relation to prematurity or birth weight [10, 11, 37]. We and others [32, 37, 58] found that having chronic medical conditions increase the risk for hospitalisation due to bronchiolitis from RSV. Jointly, these findings suggest that infants with sub-optimal health status during the peri-natal/neonatal period, reflected by prematurity or stay in neonatal intensive care unit, chronic medical conditions, comprise main risk groups for RSV morbidity during the first 2 years of life.

We found that male children had increased risk for severe RSV bronchiolitis than females, in agreement with prior findings [7, 59, 60]. Children with RSV who had pneumonia had more severe disease than those who did have pneumonia, thus confirming our [30] and others' previous reports [61].

Our study has some limitations. The burden of RSV is likely greater than reported here, since we only characterised hospitalisations for RSV bronchiolitis and some children with mild disease might be treated in the community. We used data from medical records, thus differences might exist between physicians and years in documentation of medical information. To overcome his potential limitation, we obtained and validated data across the various parts of the medical file, including laboratory results section, nursing details and follow-up, diagnoses, and hospitalisation and discharge summaries. Our study was a single-centre study; thus, the generalisability of the incidence estimates might be limited. However, our incidence estimates were comparable to those obtained in a nationwide analysis of RSV hospitalisations in Israel [25]. The control group of hospitalised children might not represent well the general population.

Our study has multiple strengths. We collected data over 10 years, thus yielding robust estimates of the incidence and risk factors for RSV, in addition to the availability of comprehensive clinical and demographic information. Moreover, our study population has good representativeness of the main population groups in Israel, the Jewish and Arab population, as well as various SES strata.

## Conclusion

The incidence of RSV disease hospitalisations constitutes a significant burden, especially in young infants. Children with chronic medical conditions, history of stay in neonatal intensive care unit at birth and those born between October and January (RSV season) were at increased risk for hospitalisation for RSV bronchiolitis. Effective preventive measures such as RSV active vaccines can reduce the risk of hospitalisations for RSV bronchiolitis among these vulnerable children.

## Data Availability

Individual-level data from this study cannot be made publically available due to legal and ethical restrictions.

## Supplementary material

For supplementary material accompanying this paper visit http://doi.org/10.1017/S0950268822000152. click here to view supplementary material

## Supplementary material

The supplementary material for this article can be found at https://doi.org/10.1017/S0950268822000152.
